# Limits of a Glycine Betaine–Derived Xenobiotic as a Trojan Horse Antimicrobial

**DOI:** 10.3390/ijms27125585

**Published:** 2026-06-20

**Authors:** Anita Dornes, Lucas Lauterbach, Jeroen S. Dickschat, Gert Bange, Erhard Bremer

**Affiliations:** 1 Faculty of Biology, Marburg University, 35043 Marburg, Germany; anita.dornes@synmikro.uni-marburg.de; 2 Faculty of Chemistry, Marburg University, 35043 Marburg, Germany; bangeg@staff.uni-marburg.de; 3 Center for Synthetic Microbiology (SYNMIKRO), Marburg University, 35043 Marburg, Germany; 4 Kekulé Institute for Organic Chemistry and Biochemistry, University of Bonn, 53121 Bonn, Germany; lauterbachlukas22@gmail.de (L.L.); dickschat@uni-bonn.de (J.S.D.)

**Keywords:** transporters, compatible solutes, osmotic stress, antibiotics, aerobic and anaerobic growth

## Abstract

Glycine betaine transport systems are widely exploited by bacteria to survive osmotic stress and represent potential entry routes for antimicrobial delivery. Here, we investigate the bactericidal glycine betaine analog Tox-GB and its uptake, intracellular fate, and antimicrobial activity in *Escherichia coli* K-12 under osmotic stress. We show that the xenobiotic enters cells via a hierarchical uptake route involving the osmotically regulated compatible solute transporters ProU and ProP, ABC- and MFS-type transporters, respectively. ProU functions as the primary high-affinity transporter at low concentrations, whereas ProP provides a secondary uptake route at somewhat higher substrate levels. Loss of either transporter confers partial resistance, while simultaneous inactivation of both systems causes full resistance, underscoring their functional redundancy and the robustness of Tox-GB import. Intracellularly, Tox-GB undergoes oxygen-dependent degradation, yielding 4-nitrobenzaldehyde and dimethylglycine. While 4-nitrobenzaldehyde contributes to toxicity under aerobic conditions, Tox-GB remains bactericidal under anaerobic conditions, indicating additional oxygen-independent mechanisms involving either the parent compound or unidentified metabolites. These findings suggest a complex intracellular fate and multifactorial mode of action. Despite initial promise as a Trojan horse antimicrobial strategy, the use of Tox-GB for practical applications faces key limitations. Resistance readily emerges via transporter inactivation, and intrinsic resistance occurs in species lacking appropriate compatible solute uptake systems. Structural constraints in glycine betaine transporters further restrict design flexibility. Osmotic regulation limits activity to specific niches, and potential host toxicity stemming from reactive metabolites raises safety concerns. Collectively, these findings highlight the mechanistic complexity and translational challenges faced by glycine betaine–derived xenobiotics as antimicrobial agents.

## 1. Introduction

Bacteria exhibit remarkable resilience to fluctuations in their ecological niches, employing coordinated genetic, biochemical, and physiological responses that minimize cellular damage and facilitate repair, thereby ensuring survival and sustained growth [1]. A common environmental challenge faced by microorganisms is exposure to sudden or prolonged increases in osmolarity or salinity. Such conditions promote water efflux from the cell, leading to dehydration and loss of turgor pressure, which can compromise cellular function and reduce ecological fitness [2,3].

To counteract the negative consequences of high-osmolarity surroundings on cellular integrity and physiology, many microorganisms accumulate compatible solutes, low molecular mass and highly water-soluble organic compounds that are non-disruptive to cellular functions [4,5,6,7,8,9]. These solutes can be synthesized or imported, with pool sizes reflecting the severity of the osmotic stress imposed onto the cell [10]. Accumulation of compatible solutes promotes water retention and re-entry to restore cellular hydration and avoids a sustained high-ionic strength cytoplasm with its negative consequences on cellular biochemistry [2,3,11]. Accumulating compatible solutes increases the internal solute concentration, helping retain or draw water back into the cytoplasm. This restores both cell volume and turgor pressure, thereby enabling growth to resume under otherwise osmotically challenging conditions [2,3,4,12,13]. Collectively, this *salt-out* cellular defense strategy [14] is conserved across the three domains of life [12,15,16,17,18], including in human cells [19].

**Figure 1:**
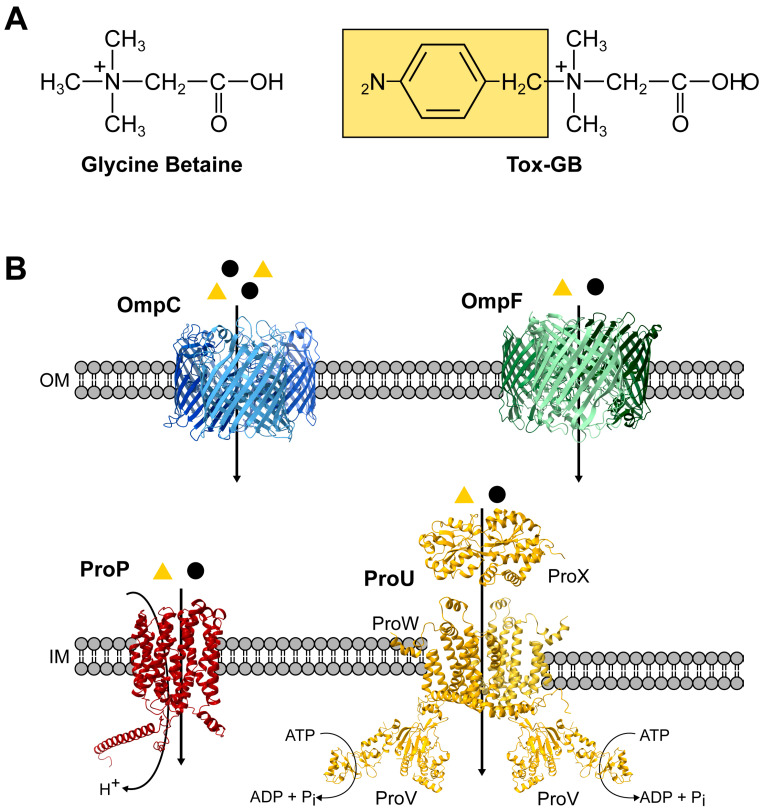
Proposed passageway of glycine betaine and its bactericidal derivative Tox-GB into osmotically stressed *E. coli* cells. (**A**) Chemical structures of glycine betaine [*N,N,N*-trimethylglycine] and Tox-GB [*N,N*-dimethyl-*N*-(4-nitrobenzyl)ammonio)acetate]. (**B**) Proposed uptake of glycine betaine (yellow triangles) and Tox-GB (black dots) in *E. coli* K-12. Under high osmolarity, glycine betaine passes the outer membrane permeability barrier via the OmpC and OmpF porins; mainly through the osmotically induced OmpC channel [48,49]. It is subsequently imported into the cytoplasm by the ProP and ProU transport systems [26]. Tox-GB likely follows the same pathway. Structural models of OmpC, OmpF, and the periplasmic binding protein ProX are based on crystal structures deposited in the Protein Data Bank (PDB). PDB IDs: 7JZ3 (OmpC), 7FDY (OmpF), and 1R9I (ProX)). Putative structures of the cytoplasmic membrane transporter ProP (AF-POCOL7-F1-v4) and the ProU components ProV (AF-P14175-F1-v4) (ATPase) and ProW (AF-P14175-F1-v4) (cytoplasmic membrane component) were predicted using AlphaFold [50], and they were retrieved from the AlphaFold Protein Structure Database (alphafold.ebi.ac.uk).

The quaternary ammonium compound glycine betaine (*N,N,N*-trimethylglycine) (Figure 1A) is a compatible solute and chemical chaperone and is probably the most widely used osmotic stress protectant in nature [4,5,12,20,21]. It can be synthesized through different combinations of enzymes in various organisms. Most pertinent to our study, it can be imported from environmental sources by microorganisms via different types of transport systems [2,3,10,12,13,22,23,24,25,26]. Transcription of the corresponding transporter genes is typically upregulated when cells experience high-osmolarity challenges [2,3,12,13,27]. Furthermore, the activity of the transporter itself is often stimulated by osmotic stress [27,28,29,30,31]. These two levels of regulation provide cells with increased import capacity for glycine betaine to counteract the outflow of water and overcrowding of the cytoplasm [2,3,11,13,32,33].

The rapid rise in antibiotic-resistance among pathogenic bacteria urgently demands innovative approaches to find new solutions for this problem, burgeoning the health systems of both developed and developing societies [34,35]. Chambers and Lever proposed in 1996 [36] that antibacterial agents built on the core chemical structure of glycine betaine (Figure 1A) could perhaps be used to fight bacterial infections [36,37]. Indeed, several glycine betaine analogs with antibacterial activity have already been synthesized [38,39,40,41,42]. Notably, Cosquer et al. reported the chemical synthesis of glycine betaine–derived compounds exhibiting either bacteriostatic or bactericidal activity [43], underscoring the potential of this compound class.

In Trojan horse strategies, microbial transport systems are exploited to mediate the uptake of antimicrobial agents that structurally mimic native substrates, thereby enabling their translocation into the cytoplasm of target cells [44]. Cosquer et al. demonstrated that glycine betaine–derived cytotoxins can be imported into bacterial cells by hijacking various types of osmotically regulated glycine betaine transport systems [39,43]. In *Escherichia coli* K-12, the binding protein–dependent ATP-binding cassette (ABC) transporter ProU and the major facilitator superfamily (MFS) transporter ProP (Figure 1B) mediate the osmotically stimulated uptake of various compatible solutes, including glycine betaine [26,45].

The Trojan horse strategy proposed by Chambers and Lever [36] has emerged as a potentially promising avenue for the development of novel antimicrobial agents [38,43,44]. Its application may be especially advantageous for targeting microorganisms adapted to high-osmolarity environments, such as those found within the human urinary tract. The elevated osmolarity of human urine (50–1200 mOsm L^−1^), combined with the presence of the osmotic stress protectant glycine betaine in it [19,36,41], and the widespread distribution of osmotically regulated glycine betaine uptake systems in bacteria [2,3,12,17,23,24,26,46], underscores the potential of glycine betaine–derived antimicrobial agents and supports their further investigation [38,40,42,43,47]. However, current knowledge regarding the physiological properties and mechanisms of action of these compounds remains limited, and the factors that may constrain their development and application as clinically relevant antimicrobial agents have not yet been comprehensively explored.

In this study, we focus on a toxic glycine betaine analog (“number 2”; hereafter referred to as Tox-GB) initially synthesized by Cosquer et al. [43]. In this compound, a 4-nitrobenzyl moiety replaces one of the three nitrogen-linked methyl groups of the head-group of glycine betaine, thus generating *N,N*-dimethyl-*N*-(4-nitrobenzyl)ammonio)acetate (Figure 1A). Clinically relevant human pathogens such as *Acinetobacter baumannii*, *Enterobacter cloacae*, *Enterococcus faecalis*, *Klebsiella pneumoniae*, *Pseudomonas aeruginosa*, *Salmonella enterica*, and *E. coli* are sensitive to Tox-GB [43].

Owing to its broad bactericidal activity against both Gram-negative and Gram-positive bacteria, Tox-GB provides a suitable model compound for evaluating the Trojan horse strategy proposed by Chambers and Lever [36]. Using *E. coli* K-12 as a genetically tractable model with well-studied glycine betaine importers (Figure 1B), we explored this concept by characterizing Tox-GB resistance–conferring mutations (Tox-GB^R^) and contextualized these findings within current knowledge of conserved glycine betaine–binding determinants in microbial transport systems [29,51,52,53,54,55,56]. Collectively, our data indicate that substantial barriers remain for the development of cytotoxic glycine betaine analogs as safe and effective antimicrobial Trojan horses, if this strategy is feasible at all.

## 2. Results

### 2.1. Import of Tox-GB Depends on the ProU and ProP Transporters, but ProU Predominates

The smuggling of glycine betaine derivatives with toxicity into bacterial cells is dependent on the occurrence, substrate specificity and type of glycine betaine transporters present in a particular microorganism [39,43]. The widely used *E. coli* K-12 laboratory strain possesses two well-studied, osmotically regulated high-affinity transporters for glycine betaine [26]. These are the binding protein-dependent ATP-binding cassette system ProU, composed of the ProV, ProW, and ProX proteins [49,57], and the single-component ProP system [45,58], a member of the major facilitator superfamily (MFS) (Figure 1B) [59]. It should be noted that *E. coli* isolates from pyelonephritis, a severe kidney infection, contain additional osmoregulatory glycine betaine importers, including the betaine–carnitine–choline transporter (BCCT) family member BetU [60,61].

**Figure 2:**
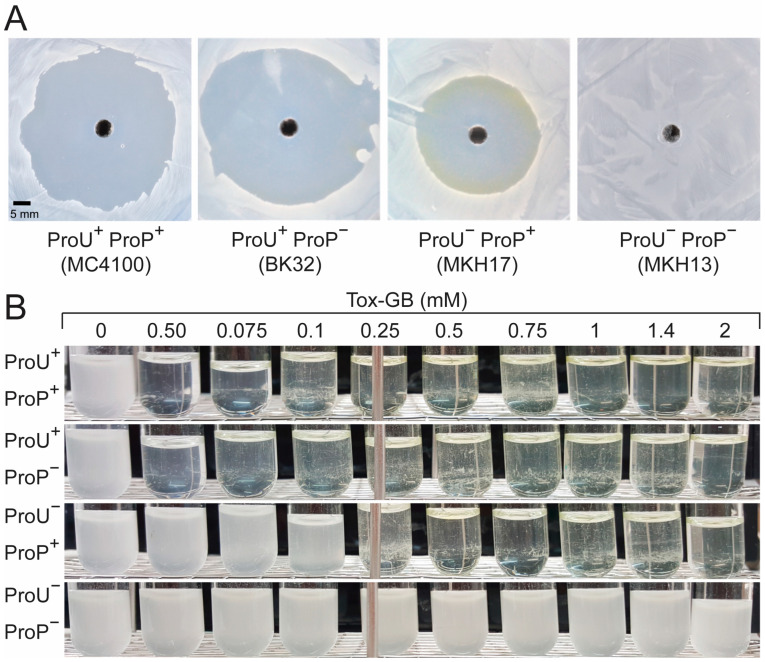
Assessing the sensitivity of *E. coli* K-12 strains to Tox-GB. (**A**) Agar diffusion assay evaluating the sensitivity of strain MC4100 and its isogenic mutants BK32, MKH17, and MKH13 [63]. Strains grown in MMA with 0.3 M NaCl were plated on MMA agar, and 70 µL of Tox-GB (100 mM stock solution) was added to a well with a 5 mm diameter punched into each plate. After two days of incubation at 37 °C, a yellow product, likely 4-nitrobenzaldehyde, was visible at the edge of the inhibition zone around the ProU^−^ ProP^+^ strain MKH17, consistent with Tox-GB breakdown [43]. The shown plates are representative of repeated Agar diffusion assays. (**B**) Growth inhibition assay of strains MC4100, BK32, MKH17, and MKH13 in liquid MMA under aerobic conditions. Pre-cultures were grown in LB medium, and 100 µL of each pre-culture was used to inoculate 2.5 mL MMA cultures containing 0.3 M NaCl and varying concentrations of Tox-GB (from 50 µM to 2 mM). Cultures were incubated at 37 °C for 24 h, after which their growth was visually inspected. The set of the growth experiments is representative of three independently prepared biological replicates.

Building on a previously reported preliminary dataset showing that the ProP and ProU systems influence the uptake of Tox-GB [43], we used an isogenic set of strains in which either ProU or ProP was functional [strains BK32 (ProU^+^ ProP^−^) or MKH17 (ProU^−^ ProP^+^)], or in which both transporters were genetically inactivated (strain MKH13) [62]. In agreement with the report of Cosquer et al. [43], the presence of either system sensitized the cells to the antibacterial activity of Tox-GB, while the simultaneous inactivation of both transporters conferred resistance in an agar plate diffusion assay (Figure 2A). The growth inhibition zone caused by Tox-GB is somewhat smaller for the ProU^−^ ProP^+^ strain MKH17 when compared to its ProU^+^ ProP^−^ counterpart strain BK32. Furthermore, we observed a distinct yellowish ring at the outer edge of the Tox-GB-induced growth inhibition zone in strain MKH17 (Figure 2A). The compound that causes the yellow color likely originates from the non-specific degradation of Tox-GB under aerobic conditions and is probably 4-nitrobenzaldehyde [43].

In addition to the agar plate diffusion assays, we also performed growth assays with the above-described set of strains in liquid Minimal Medium A (MMA) containing 0.3 M NaCl and increasing concentrations of Tox-GB (from 50 µM to 2 mM). Growth of both the wild-type strain MC4100 (ProU^+^ ProP^+^) and the ProU^+^ ProP^−^ strain BK32 was completely inhibited by 50 µM Tox-GB in the medium, whereas the ProU^−^ ProP^+^ strain MKH17 tolerated concentrations up to 100 µM Tox-GB. The ProU^−^ ProP^−^ deficient strain MKH13 was resistant to at least 2 mM Tox-GB (Figure 2B). Taken together, results from both plate- and liquid-based bioassays confirmed that Tox-GB uptake depends on the ProU and ProP compatible solute transport systems. Importantly, our data newly reveal a hierarchy in transporter activity, as ProU and ProP contributed differently to Tox-GB sensitivity of *E. coli* K-12 cells exposed to moderate osmotic stress [26].

### 2.2. Selection for Tox-GB Resistance Preferentially Yields proU Mutants

**Figure 3:**
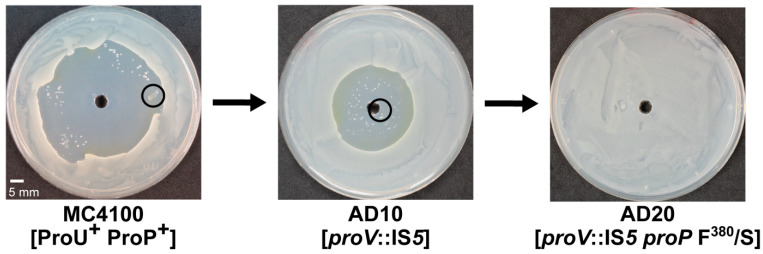
Selection of Tox-GB–resistant *E. coli* K-12 mutants. MC4100 cells grown in MMA containing 0.3 M NaCl were spread onto minimal agar plates prepared with the same components. The cells were subjected to an agar diffusion assay with Tox-GB (70 µL of a 100 mM stock solution was filled into the hole punched into the agar plate). Colonies from growth inhibition zones were isolated and purified and the DNA sequences of the *proP* gene and of the *proU* operon were determined. Shown: mutant AD10 (IS*5* insertion in *proV*), isolated from the first round of Tox-GB^R^ selection, subsequently underwent a second selection round for Tox-GB resistance, resulting in a strain (AD20) carrying an additional *proP* mutation that caused a single amino acid substitution (Phe^380^/Ser) in the ProP transporter protein.

To study the appearance and types of mutations conferring resistance to Tox-GB, we picked individual colonies from the edges of the growth inhibition zone of the wild-type strain MC4100 (ProU^+^ ProP^+^) and noted that all 11 chosen strains, which were isolated from independently grown cultures, exhibited only a reduced sensitivity to Tox-GB (Figure 3).

**Figure 4:**
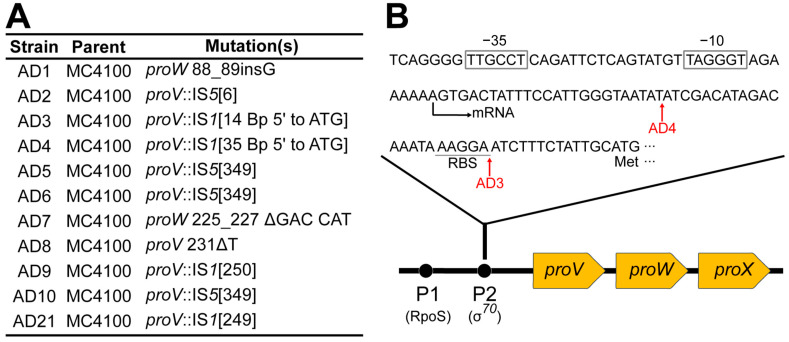
The *proU* operon is the primary target in early Tox-GB resistance mutations. (**A**) Summary of mutant derivatives of the wild-type strain MC4100 (ProP^+^ ProU^+^) isolated from the first round of selection for increased Tox-GB resistance. Mutation designations indicate the codon positions within the *proV* and *proW* genes affected by point mutations or the transposition of insertion sequences. (**B**) Strains AD3 and AD4 contain IS*1* insertions upstream of *proV*, the first gene of the *proU* operon (*proV–proW–proX*). Insertion sites are mapped relative to the -35 and -10 elements of the main, osmotically regulated *proU*-P2 promoter [64]. The predicted ribosome binding site (RBS) and start codon of *proV* are also indicated.

Since ProU and ProP serve as the sole entry routes for Tox-GB into the cytoplasm (Figure 2A), we focused our molecular analysis on their respective structural genes. Chromosomal DNA from the 11 independently selected strains was extracted, the *proU* operon and *proP* gene were individually amplified by polymerase chain reactions (PCR), and the resulting DNA fragments were sequenced. In all 11 isolates with reduced sensitivity to Tox-GB, changes in the *proU* operon were found (Figure 4A) (Table S1), while the *proP* gene was always intact. This result was somewhat unexpected, as either the ProU or ProP transporter could have been affected. However, the obtained data are consistent with our growth assays, which reveal a hierarchy in ProU and ProP transporter activity when Tox-GB is used as the substrate.

The genome of the parent *E. coli* MC4100 strain used for the selection of mutants with increased resistance against Tox-GB carries seven copies of the insertion element IS*1* and 13 copies of IS*5* [63]. Eight of the eleven isolated partial Tox-GB^R^ mutants carried insertions of these mobile elements in *proU*; four IS*1* insertions and four IS*5* insertions were recovered (Figure 4A). Six insertions had occurred in *proV*, thereby destroying the integrity of the ATPase for ProU and hence the functionality of this ABC transporter [26,49,57,65]. Two strains carried frameshifts in *proV* or *proW* (Table S1); the latter gene encodes the inner-membrane component of ProU [66] and is therefore crucial for the functioning of this transporter as well (Figure 1B).

Two IS*1* insertions into the *proU* locus had occurred upstream of the start codon of *proV*, the first gene of the *proU* operon (Figure 4B). This operon is transcribed from two osmotically regulated promoters. The upstream promoter is also stationary phase controlled, and its activity is dependent on the alternative sigma factor RpoS [67], while the main downstream positioned promoter is dependent on the house-keeping sigma factor sigma-70 [64,68]. The IS*1* element present in the *proU* 5′-untranslated region of strain AD3 has inserted 14 base pairs upstream of the *proV* ATG start codon in the vicinity of the predicted *proV* ribosome binding site (Figure 4B). The IS*1* element present in strain AD4 inserted 35 base pairs upstream of the *proV* ATG start codon and is thus present between the downstream-positioned main osmotically regulated promoter of the *proU* operon and its previously mapped transcription start site (Figure 4B) [64,68]. The reduced sensitivity of the corresponding strains to Tox-GB suggests that these two IS*1* insertions might exert a polar effect on the transcription of the *proU* operon (e.g., likely in the case of strain AD4) or might impair both transcription and translation of the *proV* gene (possibly in case of strain AD3). Thus, in these strains, the normal synthesis of the components of the ProU ABC-transporter is likely reduced or abolished, thereby explaining their decreased sensitivity to Tox-GB.

### 2.3. Exposing proU Mutants to a Second Round of Tox-GB Resistance Selection Results in proP Mutations

**Figure 5:**
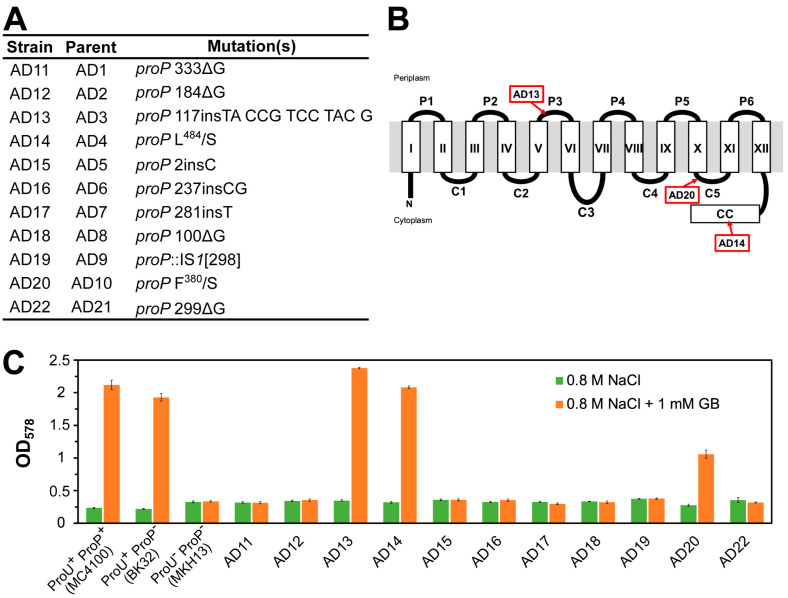
The *proP* gene is a secondary target for Tox-GB resistance mutations. (**A**) Summary of mutant derivatives isolated from a second round of selection for increased resistance to Tox-GB. Mutation designations indicate codon positions within the *proP* gene affected by point mutations or an IS*1* transposition event. (**B**) Predicted topology of the ProP transporter [45,58], a member of the major facilitator superfamily (MFS) [59]. Both the N- and C-termini of the ProP protein face the cytoplasmic side of the inner membrane. The label “CC” indicates a functionally important α-helical coiled-coil domain of ProP [45,58]. Positions of single amino acid substitutions (strains AF20 and AD14) and a four-amino-acid insertion (strain AD13) conferring resistance against Tox-GB are indicated with respect to the predicted transmembrane topology of the ProP monomer. (**C**) Osmotic stress protection assay using the compatible solute glycine betaine. Strains listed in (**A**), along with controls [strain MC4100 (ProU^+^ ProP^−^), strain BK32 (ProU^+^ ProP^−^), strain MKH13 (ProU^−^ ProP^−^)], were grown in MMA containing 0.8 M NaCl (a growth-inhibiting concentration for *E. coli* cultures grown in MMA [62]) in the presence or absence of 1 mM of the osmotic stress protectant glycine betaine. Cultures were incubated at 37 °C for 17 h. Growth data shown are representative of three independent biological replicates.

To advance our genetic analysis of the systems importing Tox-GB in *E. coli*, we separately subjected all 11 initially obtained *proU* mutants conferring partial resistance against Tox-GB to a second round of selections for Tox-GB resistance (Figure 3). In each case, mutants with full resistance against Tox-GB were obtained (Table S2), thus phenocopying the appearance of the ProU^−^ ProP^−^ strain MKH13 in the agar plate Tox-GB diffusion assay (Figure 2A). DNA sequence analysis showed that the new Tox-GB-resistant mutants all contained mutations in *proP* (Figure 5A).

ProP is a 500-residue integral cytoplasmic membrane protein that serves both as an osmosensor and osmoregulator [28,45]. As a member of the MFS of transporters [59], it exhibits the typical membrane topology with 12 transmembrane α-helices. Both the N-terminal and C-terminal domains are oriented toward the cytoplasm (Figure 5B). The extended C-terminal domain of the *E. coli* ProP protein forms an α-helical coiled-coil structure [28,45,69], which is essential for homodimer formation and proper cellular localization of the transporter protein. As such, this structure is critical for ProP’s optimal functionality [58,69].

While the movement of IS-elements into *proU* predominated the first round of Tox-GB^R^ mutant selection (Figure 4A), the second round of this selection scheme yielded *proP* mutations carrying either single-base-pair deletions or insertions (Figure 5A). Accordingly, the seven frame-shift mutations that occurred in *proP* disrupted synthesis of the full-length ProP protein, as did the only *proP*::IS*1* allele present in the mutant strain AD19 (Figure 5A). Additionally, two strains (AD14 and AD20) had point mutations that caused single amino acid substitutions [in strain AD14 (Leu^484^/Ser); in strain AD20 (Phe^380^/Ser)], while one *proP* mutation involved a four-amino-acid insertion [in strain AD13 (Ile-Pro-Ser-Tyr] in a predicted cytoplasmic loop region of ProP (Figure 5B) (Table S2). These four amino acid additions to the ProP protein chain resulted from a 12-base-pair duplication inserted into codon 117 of the *proP* gene (Table S2).

IS-elements are a major cause of spontaneous mutations in *E. coli* [70]. Hence, the preponderance (8/11) of mutations caused by the movement of IS-elements into the *proU* operon when strains with increased resistance to Tox-GB were initially selected (Figure 4A) is not unusual [71]. It is therefore somewhat surprising that among the recovered *proP* mutations in the second round of selection for Tox-GB^R^ strains, only one out of 11 studied strains carried an IS-insertion (Figure 5A). The reason for this difference is unknown but might partially be explained by the presence of a hot spot for IS*5* insertions in *proV*, the first gene of the *proU* operon (Figure 4B) (Table S1).

As expected, each of the eight strains carrying the *proP* frame-shift mutations or the *proP*::IS*1* insertion was no longer protected by glycine betaine in a growth assay of osmotically challenged *E. coli* cells (cells grown in MMA with 0.8 M NaCl and 1 mM glycine betaine) that were *a priori* defective in the ProU system (Figure 5C). Thus, the growth properties of these strains under osmotic stress conditions reflect the loss of the ProP transport activity not only for the synthetic Tox-GB compound but also for its physiologically main substrate glycine betaine [72]. Tox-GB resistance was also manifested for the three *proP* alleles, causing either single amino acid substitutions (strains AD14 and AD20) or the insertion of four amino acids (strain AD13) (Figure S1). However, the growth of the corresponding three strains was still protected from the detrimental effects of high osmolarity, at least to some extent (for instance, see the Tox-GB^R^ strain AD20), by the presence of 1 mM glycine betaine in the high-salinity medium (Figure 5C). Hence, in these three *proP* alleles, increased resistance to Tox-GB can be genetically dissociated from ProP-mediated transport of the osmotic stress protectant glycine betaine, as assessed in growth assays.

### 2.4. In Silico Evaluation of Tox-GB^R^ ProP Derivatives Retaining Glycine Betaine Transport Activity

**Figure 6:**
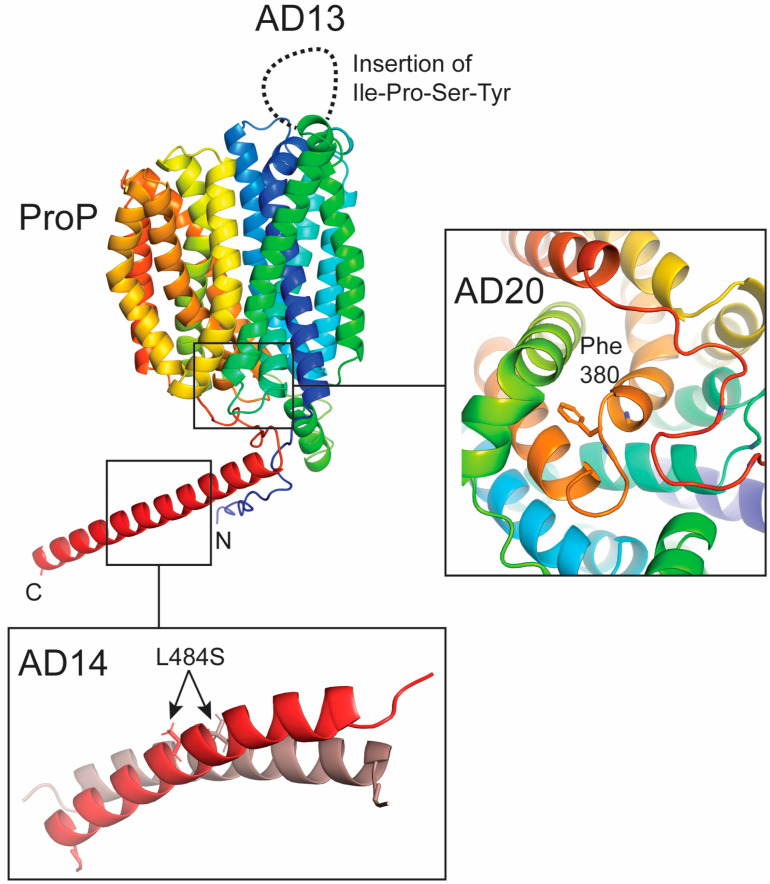
Localization and structural implications of ProP variants AD13, AD14, and AD20 identified in adaptive evolution experiments by selection for increased resistance against Tox-GB. The MFS-type transporter ProP [45,58] is shown in ribbon representation, with helices colored in a rainbow gradient from the N- to C-terminus. The model is derived from the AlphaFold Protein Structure Database (AF-P0C0L7-F1-v6) and exhibits high confidence (average pLDDT: 83.56). Insets: (i) Strain AD13, showing a four-amino-acid insertion (Ile–Pro–Ser–Tyr) located in a periplasmic loop region (dashed outline), potentially altering local loop flexibility. (ii) Strain AD14, highlighting the C-terminal helical region and the position of the Leu^484^/Ser substitution. The C-terminal extension forms a homodimeric α-helical coiled coil (red/salmon), as experimentally resolved by NMR spectroscopy (Protein Data Bank ID: 1R48) [69,73]. (iii) Strain AD20, showing a close-up of residue Phe^380^ within the transmembrane helical bundle, illustrating its local structural environment and potential role in intramolecular interactions, potentially changed through the replacement of Phe^380^ by a Ser residue.

The active ProP transporter is a homodimer [58,69]. To rationalize how the three *proP* alleles could potentially confer resistance of *E. coli* to Tox-GB while maintaining glycine betaine import activity, we examined the putative structural consequences of the two amino acid substitutions and the four-residue insertion using an AlphaFold-generated model of the ProP monomer (Figure 6).

Among the ProP variants (Figure 5B), the Leu^484^/Ser substitution in strain AD14 is particularly notable, as it might affect ProP homodimerization. ProP forms functional homodimers via coiled-coil interactions between the C-terminal α-helices [69], where Leu^484^ lies within the hydrophobic interface. Leu residues at this position typically stabilize the coiled coil through hydrophobic packing characteristic of heptad repeats. Substitution with Ser introduces a smaller, polar residue, as observed on the ProP Leu^484^/Ser variant. It might thus disrupt the hydrophobic core and weaken intermolecular packing. This is predicted to affect dimer stability or alter helix orientation, potentially impairing transporter assembly or function, given the importance of dimerization for ProP transport activity [58]. Amino acid substitutions positioned in the carboxyterminal coiled-coil segment of ProP affecting its dimerization and transporter function have already been described [69].

A similar effect is predicted for the Phe^380^/Ser substitution in strain AD20. Phe^380^ is a bulky hydrophobic aromatic residue that contributes to helix stabilization within the membrane through hydrophobic packing and helix–helix interactions. Replacement with a Ser residue removes the aromatic side chain and introduces polarity into the transmembrane region, potentially perturbing local lipid and helix interactions. This might alter helix packing or orientation, affecting the conformational dynamics required for substrate transport [58].

In strain AD13, a four-amino-acid insertion (Ile–Pro–Ser–Tyr) is located within a predicted cytoplasmic loop of ProP. Cytoplasmic loops are often involved in conformational coupling in secondary transporters. The insertion introduces residues with diverse properties, hydrophobic (Ile), conformationally restrictive (Pro), polar (Ser), and aromatic (Tyr), which may collectively alter loop geometry, flexibility, and interaction networks with adjacent domains with potentially negative consequences for ProP transporter activity.

### 2.5. Degradation of Tox-GB Leads to a Strongly Colored Yellow Compound

The osmotic stress protectant glycine betaine is metabolically inert in *E. coli* as it cannot be used as a sole carbon, energy, or nitrogen source (Figure S2A,B) while serving as an effective protectant against high-osmolarity-induced cellular challenges (Figure S2C). In contrast, Cosquer et al. [43] found that Tox-GB is unstable in aerobically grown and osmotically stressed *E. coli* cells. Tracer experiments with radioactive Tox-GB showed that it degrades over time into two metabolites, one unidentified and the other most likely 4-nitrobenzaldehyde. The latter is a yellow, chemically highly reactive, and membrane-permeable compound that is toxic to *E. coli* (Figure S3). These attributes prompted the idea that 4-nitrobenzaldehyde is responsible for the antibacterial activity of Tox-GB [43].

**Figure 7:**
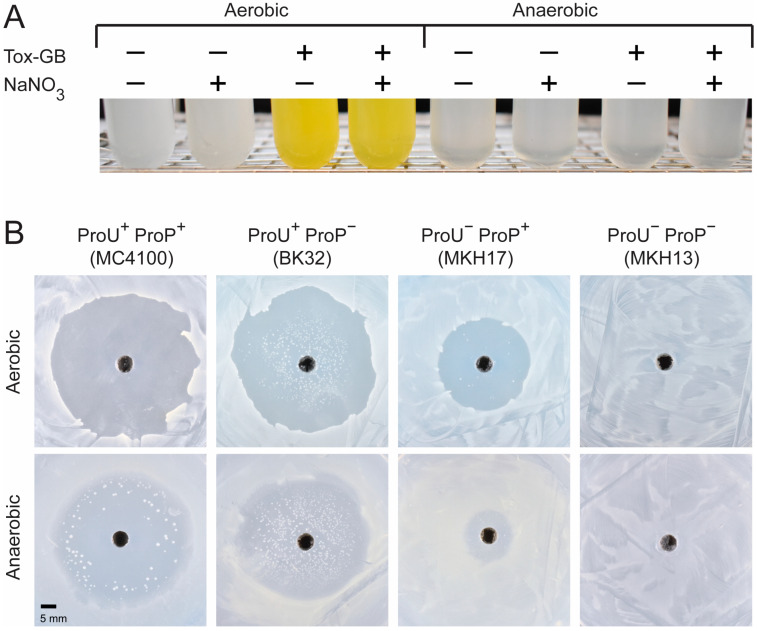
Antibacterial effects of Tox-GB and formation of 4-nitrobenzaldehyde under aerobic and anaerobic growth conditions. (**A**) Tox-GB is chemically unstable in osmotically challenged *E. coli* cells, leading to the formation of the yellow-colored compound 4-nitrobenzaldehyde [43]. To visualize its formation under aerobic and anaerobic conditions, cultures of strain MC4100 were grown in 2.5 mL MMA containing 0.3 M NaCl under either oxic or anoxic conditions until reaching an OD_578_ of about 1. Tox-GB was then added to a final concentration of 2 mM. Cultures were further incubated at 37 °C for 16 h under aerobic or for 48 h under anaerobic conditions. Formation of 4-nitrobenzaldehyde was assessed visually. In some cultures, 1 mM NaNO_3_ was added. (**B**) Agar plate diffusion assays with Tox-GB were conducted using an isogenic set of strains differing in the presence of the ProP and ProU transport systems [62]. Assays were performed under both aerobic and anaerobic conditions to evaluate Tox-GB sensitivity. The data shown for the agar diffusion assays in the presence of Tox-GB are representative of three independently prepared biological replicates.

When we grew *E. coli* cultures under aerobic conditions to an OD_578_ of about 1 and subsequently exposed the cells to Tox-GB at a concentration of 2 mM, the culture turned an intensely yellow color upon further incubation (Figure 7A). Formation of the yellow-colored compound was dependent on an increased salinity of the growth medium (Figure S4), a fact that aligns with the dependence of Tox-GB for its import into *E. coli* on the osmotically controlled ProU and ProP transporters (Figure 2A) [12,26]. Taken together, these findings suggest that Tox-GB might act under oxic conditions as a prodrug, with its antibacterial activity in *E. coli* K-12 possibly arising from intracellular degradation into toxic metabolites.

### 2.6. Tox-GB Possesses Antibacterial Activity Under Anaerobic Growth Conditions Where 4-Nitrobenzaldehyde Is Not Formed

**Figure 8:**
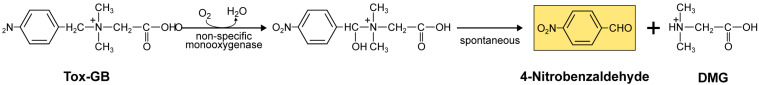
Proposal for the degradation of Tox-GB in sustained osmotically challenged *E. coli* cells grown under aerobic conditions. The proposed reaction scheme suggests that Tox-GB undergoes degradation via an initial oxidation at the reactive benzylic position. This step is likely catalyzed as a side reaction by at least one oxygen-dependent monooxygenase. The resulting unstable intermediate is a hemiaminal (carbinolammonium), which spontaneously decomposes into two products: 4-nitrobenzaldehyde, a bright, yellow-colored and membrane-permeable compound, and the ammonium salt of the compatible solute dimethylglycine (DMG).

The formation of 4-nitrobenzaldehyde from Tox-GB is thought to require at least one oxygen-dependent monooxygenase, as suggested by the reaction scheme that we propose for its formation (Figure 8). Accordingly, Tox-GB is first oxidized in the reactive benzylic position by a non-specific monooxygenase to yield a highly reactive hemiaminal intermediate (carbinolammonium) that collapses spontaneously to form the intensely yellow-colored compound 4-nitrobenzaldehyde and the ammonium salt of dimethylglycine (DMG). DMG, like glycine betaine, cannot be exploited as nutrients by *E. coli* K-12 cells (Figure S2A,B), but both compounds function as compatible solutes [74], protecting *E. coli* K-12 cells from the detrimental effects of osmotic stress (Figure S2C).

The requirement for oxygen in the proposed degradation pathway (Figure 8) prompted us to investigate whether Tox-GB retains its bactericidal activity under anaerobic conditions, where the toxic 4-nitrobenzaldehyde is not expected to form. As anticipated from the proposed degradation scheme, no yellow-colored compound was detected when osmotically stressed *E. coli* cells were exposed to Tox-GB in a liquid minimal medium in the absence of oxygen (Figure 7A). However, when *E. coli* K-12 cells were exposed to Tox-GB under anaerobic conditions, the agar plate diffusion assay still revealed antibacterial activity, indicating that this xenobiotic remains effective even in the absence of an exogenous oxygen supply (Figure 7B). The growth-inhibiting effects of Tox-GB were still dependent on intact osmotically induced ProU and ProP transporters (Figure S4), and their genetic inactivation (e.g., in strain MKH13) also leads to Tox-GB resistance under anoxic conditions (Figure 7B).

While the size of the growth inhibition zone caused by Tox-GB of the ProU^+^ ProP^−^ strain BK32 was very similar under either aerobic or anaerobic conditions, that of the ProP^+^ ProU^−^ strain MKH17 was notably reduced (Figure 7B). Increased resistance of the ProU^−^ ProP^+^ strain against Tox-GB might reflect a reduced expression of the *proP* gene in the absence of oxygen, as observed in *Shigella flexneri* [75]. Taken together, these results demonstrate that Tox-GB’s antibacterial activity under anaerobic conditions also depends on its uptake via ProU and ProP transporters but involves a different, oxygen-independent mechanism than the formation of 4-nitrobenzaldehyde.

### 2.7. Multidrug Resistance Protein EmrE Does Not Relieve the Antibacterial Activity of Tox-GB

After examining Tox-GB import via ProU and ProP transporters, we also explored whether the multidrug exporter EmrE from *E. coli* K-12 could mitigate the toxic effects of Tox-GB. EmrE is known to export various toxic, antiseptic quaternary cationic compounds [76]. In addition, Bay and Turner [77] reported that EmrE can also export the quaternary ammonium compound glycine betaine, thereby reducing its protective effects against the detrimental effects of high osmolarity on cellular physiology and growth [4,12]. We therefore wondered whether EmrE might not only facilitate the export of glycine betaine but also function as an exporter for Tox-GB, thereby mitigating its antibacterial effects. However, in the agar plate diffusion assay, the sensitivity of osmotically challenged *E. coli* cells to Tox-GB was not diminished by the presence of a plasmid-encoded *emrE* wild-type copy in comparison with strains carrying either the cloning-vector or a plasmid-based inactive version of the EmrE exporter harboring a Glu^14^ to Cys amino acid substitution [77] (Figure S5A). In the growth assay in liquid media, supernatants of cells exposed to Tox-GB and expressing the functional allele of *emrE* turned only very slightly yellowish (Figure S5B). Collectively, our results suggest that Tox-GB (or 4-nitrobenzaldehyde) does not represent a major substrate of the multidrug resistance exporter EmrE, potentially because the larger size and greater hydrophobicity of Tox-GB distinguish it from the highly water-soluble molecule glycine betaine.

## 3. Discussion

In this study, we revisited a previously proposed Trojan horse strategy [36,38,43] that exploits the osmotically regulated compatible solute transporters ProP and ProU of *Escherichia coli* K-12 [26] to facilitate the uptake of Tox-GB, a xenobiotic glycine betaine analog with bactericidal activity. By investigating Tox-GB uptake, resistance development, and antibacterial activity under both aerobic and anaerobic conditions, we provide new insights into the potential and limitations of glycine betaine–based antimicrobial approaches. When considered alongside recent structural advances in understanding glycine betaine recognition by microbial transport systems [29,51,52,54,55,56], our findings highlight the considerable challenges associated with developing such compounds into safe and effective therapeutic antimicrobials.

### 3.1. Hierarchical Import of Tox-GB by ProU and ProP Transporters

Our study advances the understanding of Tox-GB uptake into *E. coli* K-12 cells (Figure 1B). Consistent with previous findings implicating the ProU and ProP transporters in Tox-GB import [43], our genetic and physiological analyses demonstrate that these systems contribute hierarchically to uptake of the xenobiotic. The ABC-type transporter ProU preferentially mediates import at low Tox-GB concentrations, whereas the MFS-type transporter ProP provides a secondary but essential uptake route at somewhat higher concentrations (Figure 2B). This differential contribution of ProU and ProP to Tox-GB acquisition likely reflects differences in substrate affinity, specificity, and transport capacity between the two transporter systems.

Although Tox-GB involves two transporters that operate through different mechanisms and can substitute for each other, mutants resistant to Tox-GB were nevertheless obtained with relative ease (Figure 3). This finding emphasizes that targeting multiple uptake pathways in Trojan horse approaches does not necessarily prevent resistance evolution. For Tox-GB to be imported into the bacterial cytoplasm by the ProU and ProP transport systems, it must first pass through the outer membrane. The porin OmpC [33], may aid this step, as OmpC is known to allow glycine betaine to enter cells under high-osmolarity conditions [49]. However, no resistance mutations affecting porin-mediated permeability of Tox-GB were identified in our study, suggesting that altered uptake through ProU and ProP represents the primary route to resistance. Notably, while the ProP and ProU transporters are now well-established mediators of Tox-GB uptake, the cellular target(s) underlying its toxicity and its precise mode of action remain unknown and constitute an important area for future investigation.

### 3.2. Distinct Aerobic and Anaerobic Degradation Pathways of Tox-GB

A key difference between Tox-GB and the natural osmotic stress protectant glycine betaine is its intracellular instability. Under aerobic conditions, Tox-GB is likely degraded by non-specific oxygen-dependent monooxygenase activity, resulting in the formation of the chemically reactive compound 4-nitrobenzaldehyde and the non-toxic osmotic stress protectant dimethylglycine (DMG) (Figure 8). The formation of 4-nitrobenzaldehyde likely contributes to antibacterial activity through damage to cellular macromolecules and induction of stress responses. However, the persistence of antibacterial activity under anaerobic conditions, despite the absence of detectable 4-nitrobenzaldehyde formation (Figure 7A), demonstrates that this metabolite is not the sole mediator of toxicity as previously assumed [43]. Instead, intracellular accumulation of Tox-GB, or unidentified derivative(s), likely contributes to oxygen-independent antibacterial effects. Together, these findings reveal a complex intracellular fate of Tox-GB and underscore the importance of accounting for chemical stability and environmental factors, such as oxygen availability, when assessing the antimicrobial potential of glycine betaine–derived compounds.

### 3.3. Challenges for Medical Application of Tox-GB

The Trojan horse concept, based on glycine betaine analogs, initially represented an attractive strategy for selective antimicrobial delivery [36,43]. However, our findings identify several limitations for clinical development. Resistance readily emerged through inactivation of ProU and ProP, demonstrating that dependence on specific transport systems remains a major vulnerability of this Trojan horse approach [44]. Furthermore, known intrinsic resistance among bacterial species lacking suitable glycine betaine transport systems may limit the achievable spectrum of antibacterial activity [43]. Moreover, the highly conserved structural features governing glycine betaine recognition by microbial transporters, typically mediated by cation–π interactions within spatially confined aromatic cages [29,51,52,54,55,56], limit the scope for chemical modification of this compound class. The potential formation of chemically highly reactive metabolites such as 4-nitrobenzaldehyde, while contributing to antibacterial activity, may also pose risks of off-target toxicity and inflammation in human tissues. Given that glycine betaine is endogenously produced in the human kidney [19] and that human cells express glycine betaine transporters (e.g., BGT1) with physiological roles in the brain, kidney, and liver [78], cytotoxins structurally related to glycine betaine require careful evaluation of host safety and tissue distribution. Moreover, potential interference with beneficial glycine betaine–mediated pathways in human cells, including anti-inflammatory and gero-protective effects, must also be very carefully considered [79,80].

### 3.4. Conclusions

Overall, our findings establish glycine betaine–based Trojan horse strategies as a valuable proof-of-concept for transporter-guided antimicrobial delivery via osmotically induced uptake. However, their clinical potential is rather limited by transporter-dependent uptake, rapid resistance evolution, species-specific differences in transport capacity, structural constraints of substrate recognition, and potential safety concerns associated with reactive metabolites. Moreover, the requirement for osmotic induction of glycine betaine transporters for Tox-GB import restricts its cytotoxic activity to specific high-osmolarity niches, such as the human urogenital tract [18,37]. Collectively, substantial optimization of compound design, selectivity, and safety will be required before glycine betaine–derived Trojan horse antimicrobials can seriously be considered for medical application.

## 4. Materials and Methods

### 4.1. Chemicals

Glycine betaine, dimethylglycine (DMG), 4-nitrobenzaldehyde, and acetone were purchased from Sigma-Aldrich (Taufkirchen, Germany). Bromomacetic acid and *n*-butanol were purchased from Alfa Aesar (Karlsruhe, Germany). Diisopropylethalamine, acetic acid, and silica gel for column chromatography (0.04–0.06 nm) were purchased from Acros Organics (Geel, Belgium). POLYGRAM SIL G UV254 TLC plates were supplied by Macherey-Nagel (Düren, Germany). NMR spectra were recorded on a Bruker Avance I 500 MHz spectrometer (Bruker, Hamburg, Germany).

### 4.2. Synthesis of N,N-Dimethyl-N-(4-nitrobenzyl)ammonio)acetate (Tox-GB)

Chemical synthesis of *N,N*-dimethyl-*N*-(4-nitrobenzyl)ammonio)acetate (=Tox-GB) followed a previously reported procedure [81] with some modifications (Figure S6). To a mixture of *N,N*-dimethyl-1-(4-nitrophenyl)methanamine (2.0 g, 11.1 mmol, 1.0 eq) in acetonitrile (60 mL), *N,N*-diisopropylethylamine (2.9 mL, 16.6 mmol, 1.5 eq) and bromoacetic acid (1.75 g, 12.2 mmol, 1.1 eq) were added. The mixture was stirred at room temperature for 20 h and was then concentrated under reduced pressure and partitioned between water and methylene chloride. The organic phase was extracted with water, and the combined aqueous phases were washed with methylene chloride and concentrated under reduced pressure. The residue was purified using silica gel column chromatography (from Acros Organics) using an *n*-butanol/acetic acid/water mixture (5:1:1) as the eluent. Pooled fractions were concentrated under reduced pressure to achieve pure *N,N*-dimethyl-*N*-(4-nitrobenzyl)ammonio)acetate (=Tox-GB) (2.21 g, 9.8 mmol, 89% yield). The obtained compound was characterized by thin-layer chromatography (TLC), ^1^H-NMR and ^13^C-NMR spectroscopy. TLC (on POLYGRAM SIL G UV254 TLC plates): *R*_f_ (*n*-butanol/acetic acid/water, 5:1:1) = 0.31. ^1^H-NMR (500 MHz, D_2_O): *d* = 8.29 (d, ^3^*J*_H,H_ = 8.1 Hz, 2 x CH), 7.71 (d, ^3^*J*_H,H_ = 8.1 Hz, 2 x CH), 4.86 (s, CH_2_), 3.47 (s, CH_2_), 3.21 (s, 2 x CH_3_) ppm. ^13^C-NMR (125 MHz, D_2_O): *d* = 169.0 (C_q_), 148.9 (C_q_), 134.5 (C_q_), 134.0 (2 x CH), 124.2 (2 x CH), 65.3 (CH_2_), 63.2 (CH_2_), 51.3 (2 x CH_3_) ppm.

### 4.3. Bacterial Strains and Plasmids

The *E. coli* K-12 laboratory strain MC4100 [63] was used throughout this study as the wild-type strain, as it possesses intact ProP and ProU glycine betaine import systems [26,63]. Strains BK32 [Δ(*proP*)2)], MKH17 [Δ(*proU::spc^R^*)608)], and MKH13 [Δ(*proP*)2) Δ (*proU::spc^R^*)608)] are derivatives of MC4100 and carry gene disruption mutations of either *proP*, of the *proU* operon, or of both loci [62].

Plasmids carrying the *emrE* wild-type gene (pEmrE), or the gene for its inactive mutant derivative EmrE-E^14^/C [present on plasmid pEmrE-E14C] were kindly provided by Dr. Raymond J. Turner (University of Calgary; Canada) [77]. The expression vector pMS119EH (P*tac*/*lacI*^q^) [82] is the plasmid backbone that had been used to construct the pEmrE and pEmrE-E14C plasmids; it carries an ampicillin resistance determinant (Amp^R^). The pEmrE^+^ and pEmrE-E14C plasmids express the *emrE* alleles under the transcriptional control of the leaky IPTG-inducible *tac*/*lacI*^q^ hybrid promoter [82]. Plasmid pEmrE-E14C expresses a non-functional EmrE protein in which the codon for the active-site glutamate residue was replaced by a codon for cysteine; this change was generated through site-directed mutagenesis [77]. The expression vector pMS119EH and the plasmids carrying *emrE* were transformed [83] into the *E. coli* K-12 mutant strain AD21, a derivative of strain MC4100 [63], that is partially resistant to Tox-GB due to a *proV*::IS*1* insertion mutation (Figure 4A). The possible function of EmrE for Tox-GB export was tested without or by adding IPTG (1 mM) to the cultures of strain AD21 (MC4100 *proV*::IS*1*) carrying either the pMS119EH, pEmrE, or pEmrE-E14C plasmids.

### 4.4. Growth Media and Culture Conditions

*E. coli* strains were maintained on LB-agar plates [84]. Tests for sensitivity/resistance against Tox-GB were conducted either on agar plates prepared with the components of Minimal Medium A (MMA) [84] or in MMA liquid cultures. Unless stated otherwise, cultures were grown in test tubes filled with 5 mL medium. Glucose (0.5%; *w*/*v*) was used throughout this study as an energy and carbon source for *E. coli* cultures grown in minimal media. The salinity of agar plates, or that of liquid cultures, was increased through the addition of NaCl from a 5 M stock solution as detailed in the individual experiments. When required, 100 µg mL^−1^ ampicillin was added to cultures of strains carrying pMS119EH-derived plasmids. The use of glycine betaine and DMG as sole carbon and energy sources by *E. coli* was assessed by replacing glucose (28 mM) as the carbon and energy source in MMA with 33 mM glycine betaine and 42 mM DMG. The use of these solutes as sole nitrogen sources by *E. coli* was tested by replacing the ammonium source [(NH_4_)_2_SO_4_] present in MMA with 30 mM of either glycine betaine or DMG [74].

To test the potential role of the EmrE exporter in alleviating Tox-GB-mediated growth inhibition and/or export of 4-nitrobenzaldehyde, strains carrying the vector pMS119EH (P*tac*/*lacI*^q^), pEmrE^+^, or pEmrE-E14C were initially grown in test tubes containing 3 mL LB medium, and 100 µL of these cultures was used to inoculate the main culture (3 mL MMA supplemented with 0.3 M NaCl). The cultures were grown to an OD_600_ of 1, at which point 2 mM Tox-GB (final concentration) and 1 mM IPTG were added. The cultures were then incubated overnight at 37 °C with shaking. The formation of the yellow-colored 4-nitrobenzaldehyde by the cultures and its presence in the culture supernatant were assessed through visual inspection (Figure S5B).

To grow various *E. coli* strains in liquid medium anaerobically, Hungate tubes (Dunn, Asbach, Germany) with rubber stoppers were filled with degassed MMA, and the remaining air in the tubes was replaced with nitrogen–CO_2_ gas. Hungate tubes with a total volume of 16 mL were used for the experiments. Each tube contained 5 mL of medium, and the gas phase consisted of N_2_/CO_2_ (80:20, *v*/*v*). NaNO_3_ was added at a final concentration of 1 mM to some of the cultures to provide an additional electron acceptor under anaerobic growth conditions. NaNO_3_ may reduce the accumulation of acidic fermentation products and thereby may help to prevent excessive acidification of the medium. However, the presence or absence of NaNO_3_ did not affect the outcome of the experiment (Figure 7A). For the growth experiment under anaerobic conditions, the medium was inoculated with 0.1 mL of a pre-culture grown under aerobic conditions in MMA containing additional 0.3 M NaCl. The cultures were not shaken, and they were incubated at 37 °C until they reached an OD_578_ of about 1. At this point, 2 mM of Tox-GB (final concentration) from a 100 mM stock solution (in 50 mM Tris-HCl; pH 7.5) were added to the cultures, and these were subsequently further incubated at 37 °C without shaking overnight. The cultures were then visually inspected for growth and the formation of the yellow-colored 4-nitrobenzaldehyde.

A similar procedure was used to grow cells exposed to Tox-GB under aerobic conditions. For these experiments, cultures of the *E. coli* K-12 strain MC4100 were grown in MMA containing 0.3 M NaCl for about 16 h in the absence or presence of Tox-GB (2 mM Tox-GB; final concentration). The cells were then pelleted in a tabletop centrifuge, and both the supernatant and the cell pellet (Figure S4) were visually inspected for the formation of colored compounds. A control culture of MC4100 was grown and treated in the same way, except that the culture was not exposed to Tox-GB.

When *E. coli* strains were assessed for sensitivity against Tox-GB in an agar plate diffusion assay under anaerobic conditions, cultures were grown aerobically in MMA containing 0.3 M NaCl until they reached an OD_578_ of about 2. Subsequently, 0.1 mL of these pre-cultures were spread on MMA-based agar plates that contained 0.3 M NaCl. The cells were then subjected to an agar plate diffusion assay with Tox-GB; the agar plates were incubated at 37 °C in an anaerobic jar for 48 h using the BD GasPak^TM^ system from Fischer Scientific (Schwerte, Germany).

### 4.5. Growth Assays Assessing the Antibacterial Activity of Tox-GB in an Isogenic Set of E. coli Mutants with Defects in the ProP and/or ProU Transporters

To assess the sensitivity of *E. coli* K-12 strains against Tox-GB, an isogenic set of strains constructed in the genetic background of strain MC4100 (ProP^+^ ProU^+^) was used [62,63]. In these strains, the glycine betaine transport systems ProP and ProU were either individually genetically inactivated [strain BK32 (ProU^+^ ProP^−^) or strain MKH17 (ProU^−^ ProP^+^)] or both transporters were not functional (strain MKH13) [62]. Agar diffusion tests with Tox-GB were conducted as follows: cultures of the various strains were [62,63] pre-grown at 37 °C in MMA containing 0.3 M NaCl until they reached an OD_578_ of about 2. Then, 100 µL of the cultures were spread onto an MMA agar plate that also contained 0.3 M NaCl. Using the back of a sterile Pasteur pipette, a hole with a diameter of about 5 mm was cut out of the agar plate, and 70 µL of a Tox-GB solution (100 mM dissolved in 50 mM Tris-HCl; pH 7.5) was pipetted into the hole. After incubation of the agar plates for 24 h at 37 °C, the size of the growth inhibition zone around the hole filled with the Tox-GB solution was visually assessed.

Growth inhibition of strains MC4100, BK32, MKH17, and MKH13 by Tox-GB was also assessed in liquid MMA under aerobic conditions. Pre-cultures were grown in LB medium, and 100 µL of these cultures were then used to inoculate 2.5 mL cultures of MMA containing 0.3 M NaCl and various concentrations of Tox-GB (from 50 µM to 2 mM). The cultures were grown at 37 °C for 24 h, and the OD_578_ was then determined. The sensitivity of the strains tested against Tox-GB was determined in three independently prepared replicates.

Tox-GB is chemically unstable in *E. coli*, and its degradation in osmotically challenged cultures yields, among other compounds, the yellow-colored 4-nitrobenzaldehyde [43]. To visualize the formation of this compound under oxic and anoxic growth conditions, a culture of strain MC4100 was first grown in LB medium for 3 h. Then, 100 µL of this pre-culture was used to inoculate 2.5 mL of MMA medium containing 0.3 M NaCl, and these cultures were then separately grown under aerobic and anaerobic conditions until they reached an OD_578_ of about 1. At this time point, Tox-GB was added to the cultures at a final concentration of 2 mM. The cultures were further incubated at 37 °C either for 16 h (for the aerobic cultures) or for 48 h (for the anaerobic cultures). The formation of the yellow-colored 4-nitrobenzaldehyde was visually assessed.

### 4.6. Selection of E. coli Mutant Strains with Increased Tolerance Against Tox-GB

To select mutants with increased tolerance against Tox-GB, 0.1 mL of 11 independently grown cultures of the ProP^+^ ProU^+^ strain MC4100, pre-grown in MMA containing 0.3 M NaCl, were spread onto MMA plates containing 0.3 mM of NaCl. The *E. coli* cells were subjected to an agar diffusion test with Tox-GB [70 µL of a Tox-GB solution (100 mM stock solution in 50 mM Tris-HCl; pH 7.5) filled into a hole (5 mm diameter) punched into the agar plate)]. After two days of incubation of the agar plates at 37 °C, a single colony was picked from the growth inhibition zone. These strains were purified by re-streaking them twice on MMA plates without additional NaCl and no Tox-GB; subsequently, they were subjected again to an agar diffusion test with Tox-GB (on MMA-based minimal medium agar plates containing 0.3 M NaCl) to visually assess their degree of sensitivity/resistance against the xenobiotic. Each of the initially isolated mutants showed a decreased size of the growth inhibition zone, but none of them was truly resistant to Tox-GB (see Figure 3 for an example). A collection of 11 independently isolated mutants obtained in the first round of selection for increased resistance against Tox-GB was then used for a second round of selection for derivatives with further increased levels of Tox-GB resistance (Figure 3) using the above-described agar diffusion test and the same amount of Tox-GB used for their original isolation. After two rounds of purification of the strains on MMA plates without additional NaCl and no Tox-GB, they were subjected again to resistance/sensitivity tests against Tox-GB on MMA agar plates containing 0.3 M NaCl; these strains were found to be resistant to Tox-GB.

### 4.7. Targeted DNA Sequence Analysis of proU and proP Genes Present in Tox-GB^R^ Mutants

To determine the type of mutations in the *proP* gene or the *proU* operon causing increased resistance against Tox-GB, chromosomal DNA of corresponding strains was prepared according to Marmur [85]. A 5 mL culture of a given strain was inoculated with a single colony picked from an LB-agar plate and was grown for 3 h at 37 °C. The prepared chromosomal DNA was resuspended in 100 µL double-distilled water and subsequently used as the starting material for targeted PCRs to amplify either the entire *proP* gene or the *proU* operon (*proV-proW-proX*). For PCRs targeting the amplification of the *proP* gene and of the *proU* operon, a 50 µL reaction volume and 50 ng of isolated chromosomal DNA were used as the template using DNA-primers (purchased from ThermoScientific (Dreieich, Germany) as specified in Table S3. A PCR system (PCR-Cycler Advanced Primus 25) from PEQLAB (Erlangen, Germany) was used to prepare the targeted genomic *proP* and *proU* DNA-segments. DNA sequence analysis of the amplified regions was carried out by Eurofin MWG (Ebersberg, Germany) using the procedure of Sanger et al. [86] and a set of custom-synthesized DNA primers (Table S3). The DNA sequences obtained from the *proP* gene and the *proU* operon from the various Tox-GB^R^ mutants were compared to the genome sequence of the *E. coli* K-12 laboratory strain MC4100 (GenBank/EMBL/DDBJ accession number CP001396) [63].

### 4.8. Osmotic Stress Protection Assays

To assess the ability of MC4100 and its derived *proU proP* Tox-GB^R^ mutant strains to withstand osmotic stress either in the absence or the presence of glycine betaine [62], pre-cultures were prepared in LB medium and grown overnight. Then, 0.1 mL of this culture was used to inoculate a culture grown in MMA containing 0.3 M NaCl to pre-adapt the cells to increased salinity. This culture was grown until it reached an OD_578_ of about 1. Using cells from this pre-culture, the main cultures (20 mL of MMA with 0.8 M NaCl), either in the absence or the presence of 1 mM glycine betaine, were inoculated to an OD_578_ of about 0.1 and were then incubated at 37 °C in 100 mL Erlenmeyer flasks set in a rotating water bath; they were typically grown for 17 h. Growth yield of the cultures was determined by measuring their optical density (OD_578_) in a photo-spectrometer. Growth of the *E. coli* K-12 strain MC4100 in MMA that contains 0.8 M NaCl is strongly inhibited, while the addition of 1 mM glycine betaine to the high-osmolarity cultures rescues growth, a property that is dependent on either the ProP and ProU compatible solute transporters [62].

## Data Availability

The original contributions presented in this study are included in the article/Supplementary Material. Further inquiries can be directed to the corresponding author.

## Supplementary Materials

The following supporting information can be downloaded at: https://www.mdpi.com/article/10.3390/ijms27125585/s1.
