# Supplementary material for: Limits of a Glycine Betaine–Derived Xenobiotic as a Trojan Horse Antimicrobial
Source: Int J Mol Sci. 2026 Jun 20;27(12):5585. doi: 10.3390/ijms27125585 (PMC13300293; doi:10.3390/ijms27125585)
Supplement: Supplementary file 1 [file ijms-27-05585-s001.zip › ijms-4373049-supplementary.pdf]

Supplementary Material for:

Smuggling a glycine betaine-derived toxic xenobiotic into osmotically stressed *Escherichia coli* cells

Anita Dornes[1,2,3], Lucas Lauterbach[4], Jeroen S. Dickschat[4], Gert Bange[2,3], and Erhard Bremer[1,3,*]

[1]Faculty of Biology, Marburg University, Marburg, Germany
[2]Faculty of Chemistry, Marburg University, Marburg, Germany
[3]Center for Synthetic Microbiology (SYNMIKRO), Marburg University, Marburg, Germany
[4]Kekulé Institute for Organic Chemistry and Biochemistry, University of Bonn, Germany

Correspondence:

Erhard Bremer
bremer@staff.uni-marburg.de

Running title: Antibacterial activity of a xenobiotic

For correspondence during the reviewing and editorial process please contact:
Prof. Dr. Erhard Bremer, Marburg University, Center for Synthetic Microbiology (SYNMIKRO), Karl-von-Frisch Strasse 14, D-35043, Marburg, Germany. Phone: (+49)-6421-2821529; E-Mail: bremer@staff.uni-marburg.de

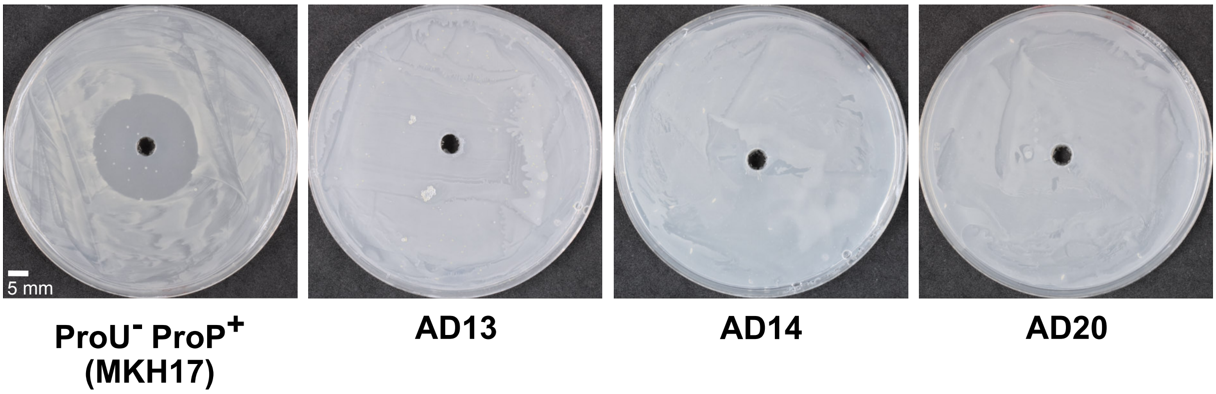

FIG S1 Resistance of ProP variants to Tox-GB. Agar plate diffusion assays were performed with strains carrying *proP* alleles that encode full-length ProP proteins in a Δ*proU* background of the *E. coli* strain MC4100 [1, 2]. Strain AD13 expresses a ProP protein with a four amino acid insertion (Ile-Pro-Ser-Tyr) in an extracellular loop. Strain AD14 synthesizes a ProP protein with a Leu484→Ser substitution, and strain AD20 carries a ProP Phe380→Ser mutation. These mutants all exhibit resistance to Tox-GB but still allow osmoprotection (at least to a certain degree; see Fig. 5C) by 1 mM glycine betaine under otherwise growth-inhibiting conditions (MMA with 0.8 M NaCl).

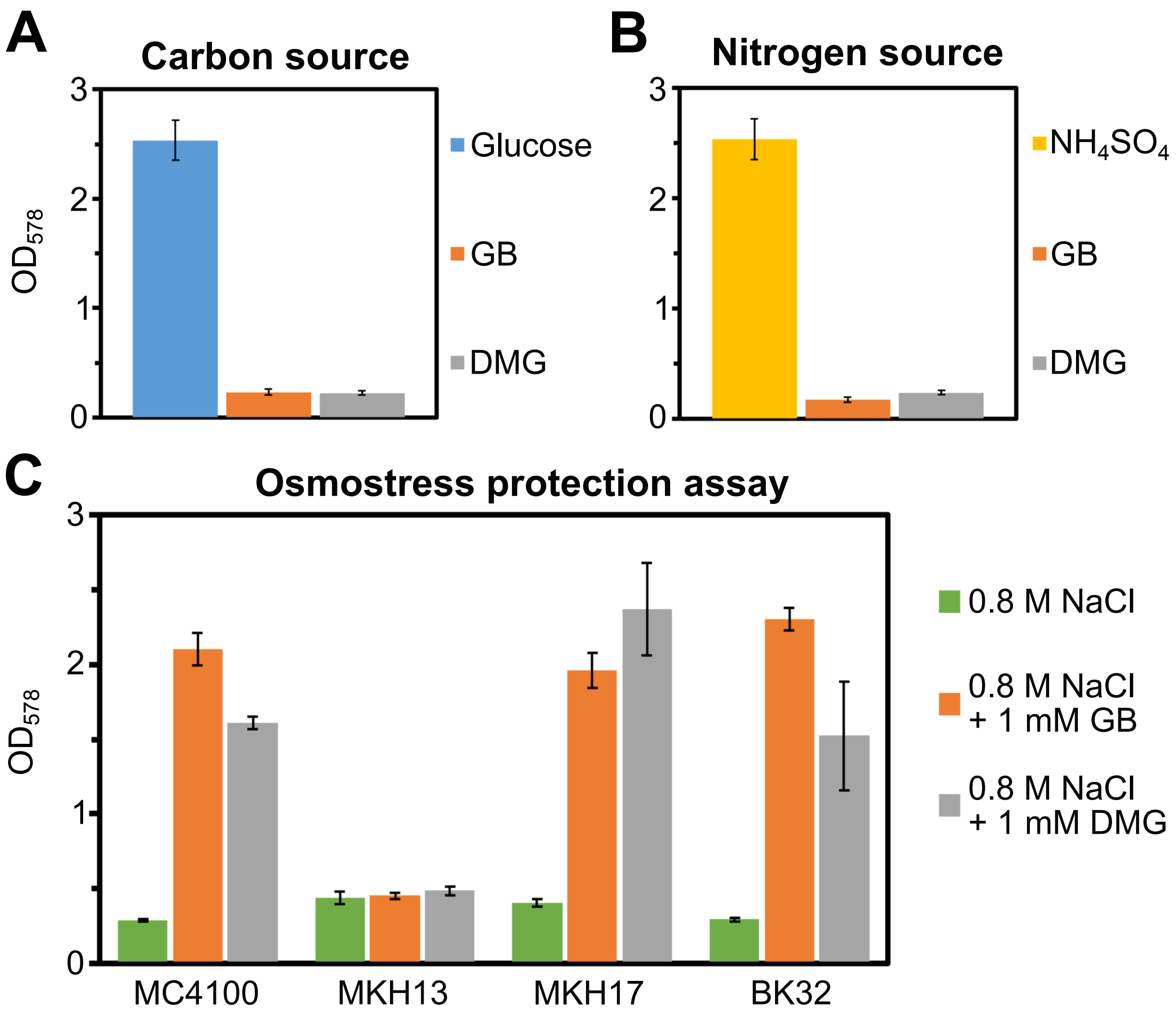

FIG S2 Dimethylglycine (DMG) functions as an osmostress protectant but cannot be used as a nutrient. Hydrolysis of Tox-GB in osmotically stressed *E. coli* cells yields toxic 4-nitrobenzaldehyde and the compatible solute dimethylglycine (DMG) [3, 4]. Growth assays in MMA assessed DMG utilization as a sole carbon and energy source (A) or as a sole nitrogen source (B). The use of glycine betaine and DMG as sole carbon and energy sources by *E. coli* was assessed by replacing glucose (28 mM) as the carbon and energy source in MMA with 33 mM glycine betaine and 42 mM DMG. The use of these solutes as sole nitrogen sources by *E. coli* was tested by replacing the ammonium source [$(NH_4)_2SO_4$] present in MMA with 30 mM of either glycine betaine or DMG. (C) At low concentrations (1 mM), both GB and DMG protected MMA-grown *E. coli* cultures exposed to 0.8 M NaCl from the detrimental effects of high osmolarity [1]. The isogenic strain set included MC4100 ($ProP^+$ $ProU^+$), MKH13 ($ProP^-$ $ProU^-$) MKH17($ProP^+$ $ProU^-$), and BK32($ProP^-$ $ProU^+$) [1]. Growth yields show two data derived from at least two independent cultures grown at 37 °C for 17 h.

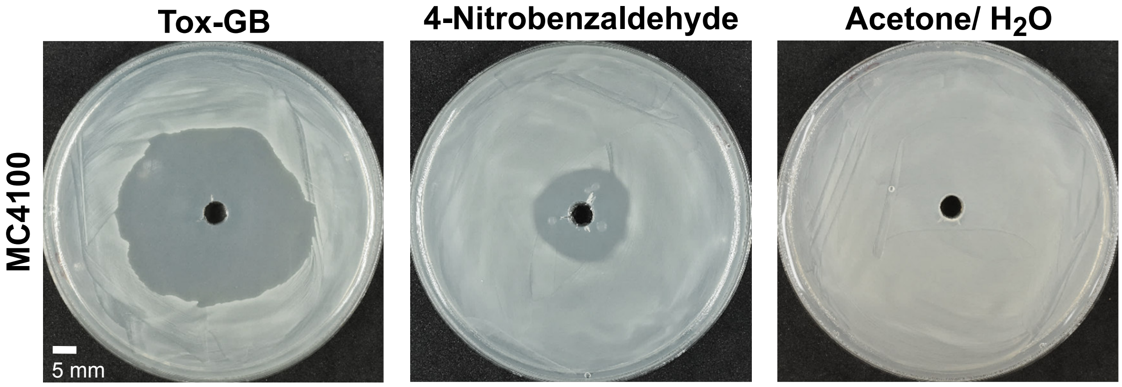

FIG S3 4-Nitrobenzaldehyde is toxic for *E. coli* cells. Cells of pre-grown cultures of strain MC4100 ($ProP^+$ $ProU^+$) were spread on MMA minimal medium agar containing 0.8 M NaCl and were tested by agar diffusion assay with Tox-GB, 4-nitrobenzaldehyde, or solvent controls. The agar plates were incubated at 37° C for about 48 h. For each of the compounds, 70 µl were filles in the hole cut-out in the agar plate. 4-nitrobenzaldehyde was solved into 1:1 solution of acetone and water. For the solvent control, a 1:1 solution of acetone and water was prepared. Tox-GB was dissolved in 50 mM Tris-HCl (pH 7.5).

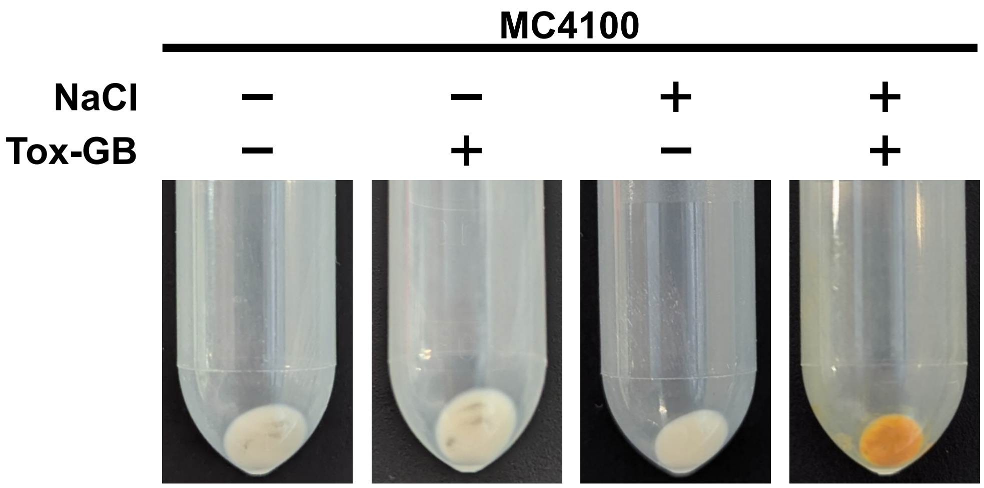

FIG S4 4-Nitrobenzaldehyde accumulates inside osmotically challenged *E. coli* cells. Cultures of strain MC4100 ($ProP^+$ $ProU^+$) was grown in MMA minimal medium, either without (-) or with (+) 0.3 M NaCl. The inclusion of NaCl in the growth medium leads to induction of *proP* and *proU* expression [5, 6]. At an OD_{578} ≈ 1, cultures were either left untreated (–) or supplemented with 2 mM Tox-GB (+). Growth was continued for about 16 h, and cells were collected by centrifugation.

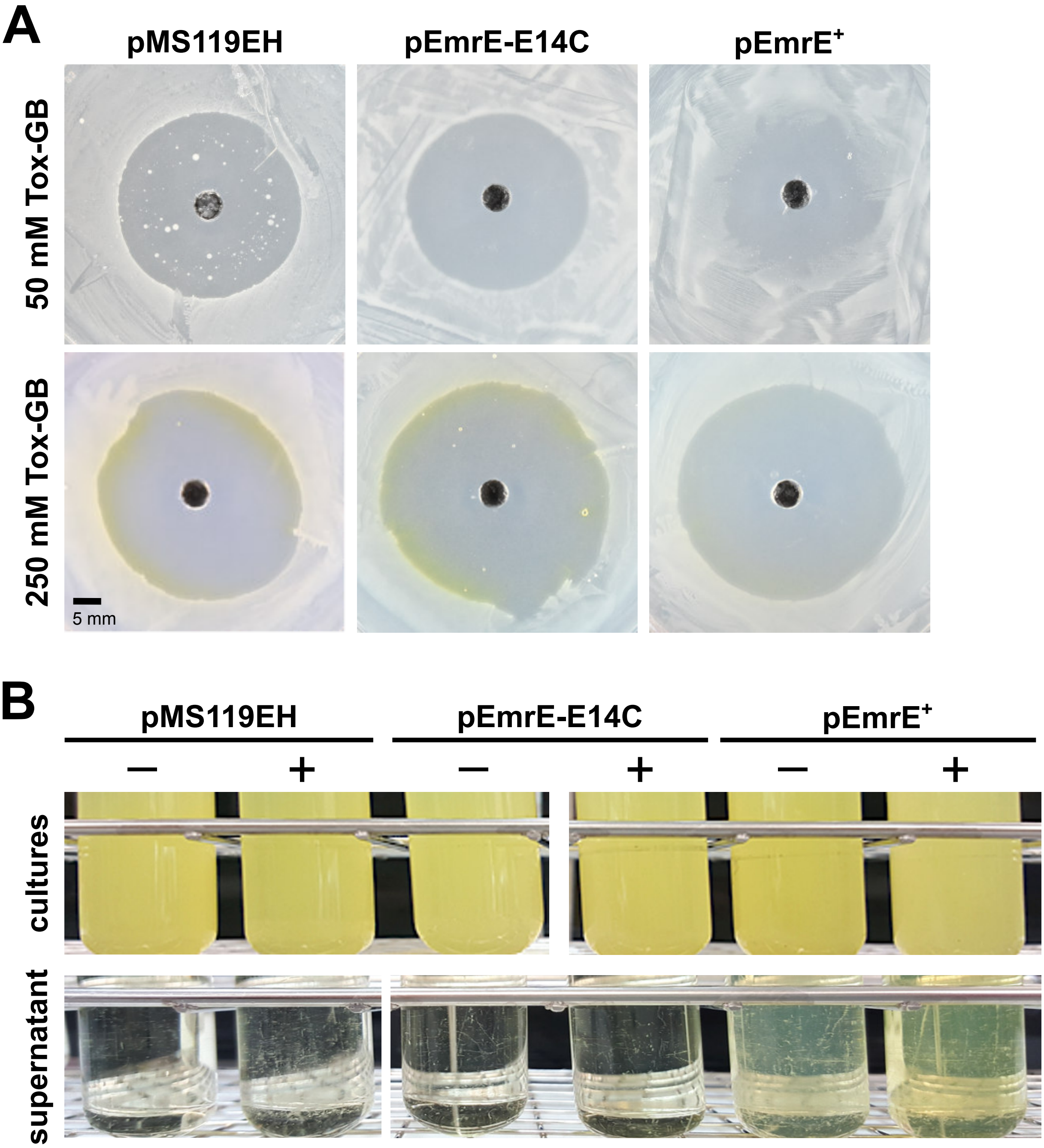

FIG S5 The multidrug exporter EmrE has limited effect on Tox-GB–mediated growth inhibition and export of 4-nitrobenzaldehyde. (A) Strain MC4100 ($ProP^+$ $ProU^+$) carrying either the empty vector (pMS119EH), wild-type *emrE* gene ($pEmrE^+$), or a *emrE* allele encoding an inactive EmrE exporter (pEmrE-E14C; Glu14→Cys substitution) [7] were spread on MMA agar plates containing 0.3 M NaCl and were tested by agar diffusion assay with varying Tox-GB concentrations. Plates were incubated at 37 °C for 24 h. (B) MC4100 ($ProP^+$ $ProU^+$) cultures were grown in MMA with 0.3 M NaCl, and when they had reached an $OD_{578} \approx 1$, they were exposed to 2 mM Tox-GB. Growth was continued for about 16 h. IPTG induction (+, 1 mM) was used to drive enhanced expression of plasmid-borne *emrE* from the *lac* promoter. Cultures marked with (-) were grown in the absence of IPTG.

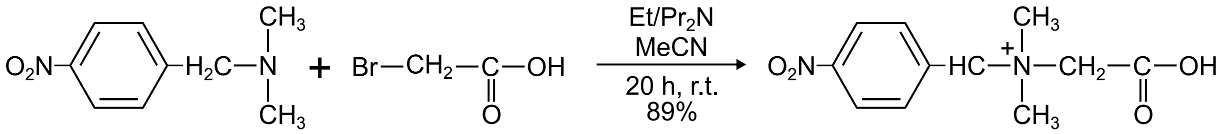

FIG S6 Synthesis of *N,N*-dimethyl-*N*-(4-nitrobenzyl)ammonioacetate (Tox-GB). A solution of *N,N*-dimethyl-1-(4-nitrophenyl)methanamine (2.0 g, 11.1 mmol, 1.0 eq) in acetonitrile (60 mL) was treated with *N,N*-diisopropylethylamine (2.9 mL, 16.6 mmol, 1.5 eq) and bromoacetic acid (1.75 g, 12.2 mmol, 1.1 eq). The reaction mixture was stirred at room temperature for 20 h, concentrated under reduced pressure, and partitioned between water and methylene chloride. The organic phase was extracted with water, and the combined aqueous layers were washed with methylene chloride and concentrated under reduced pressure. Purification of Tox-GB was achieved by silica gel column chromatography using *n*-butanol/acetic acid/water (5:1:1) as the eluent. The synthesis scheme for Tox-GB followed a previously reported protocoll [8] with minor modifications.

Table S1. *proU* mutations leading to increased resistance against Tox-GB[a]

Strain	Mutations and insertion site [5' to 3'] of IS-elements
**AD1**<br>*proW* 88_89insG	GTT-CCG^{90} → GTT [G] CC^{90}-G
**AD2**<br>*proV*::IS*5* [6]	ATT-AAA-TTA-G^{6} [GGA..**IS*5***..TCC] TTA-GAA^{6}-ATT
**AD3**<br>*proV*::IS*1* [14 Bp 5' to the ATG of *proV*]	ACAAATAAAGG [GGT..**IS*1***..ACC] AAATAAAGGAA
**AD4**<br>*proV*::IS*1* [35 Bp 5' to the ATG of *proV*]	ATATCGACATA [GGT..**IS*1***..ACC] ATCGACATAGA
**AD5**<br>*proV*::IS*5* [349]	CCG-TTA-G^{349} [GGA..**IS*5***..TCC] TTA-GCA-GTC
**AD6**<br>*proV*::IS*5* [349]	CCG-TTA-G^{349} [GGA..**IS*5***..TCC] TTA-GCA-GTC
**AD7**<br>*proW* [225_227] Δ(TGACCA)	CGT-CTG-ACC-ATT^{227} → CGT-CΔ(TG-ACC-A)TT^{227}
**AD8**<br>*proV* [231] Δ(T)	CGT-ATT^{231} → CGT-AΔ(T)T^{231}
**AD9**<br>*proV*::IS*1* [250]	GGC-ACA-CCG^{249} [GTG..**IS*1***..ACC] GC-ACA-CCG-
**AD10**<br>*proV*::IS*5* [349]	CCG-TTA^{348}-G [GGA..**IS*5***..TCC] TTA-GCA-GTC
**AD21**<br>*proV*::IS*1* [249]	GTC-GGC-ACA-CC^{249} [GGT..**IS*1***..ACC] C-GGC-ACA-CCG

[a]The identified mutations and insertion sites of IS*1* and IS*5* elements are located within the *proU* operon (*proV*–*proW*–*proX*) of the ProP^{+} ProU^{+} parent strain MC4100 [5]. The affected codons in the *proV* and *proW* genes [9, 10], either carrying mutations or hosting IS-element insertions, are indicated. Duplicated sequences flanking the IS*1* or IS*5* insertion sites are underlined. Ins: insertion; Δ: deletion.

Table S2. *proP* mutations leading to increased resistance against Tox-GB[a]

Strain	Mutations and insertion sites [5‘ to 3‘] of an IS*1*-element
**AD11** *proP* [333] Δ(G)	AGT-GTT^{333} → AGT-Δ(G)TT^{333}
**AD12** *proP* [184] Δ(G)	GTG-GTG^{184} → GTG-GTΔ(G)^{184}
**AD13** *proP* [117] ins (TACCGTCCTACG)	GGC-TTA-A **[**TA-CCG-TCC-TAC-G**]** TA^{117}-CCG
**AD14** *proP* [L^{484}/S]	TTG^{484}/ TCG
**AD15** *proP* [2] ins (C)	ATG **[**C**]** CTG^{2}
**AD16** *proP* [237] ins (GC)	CAG-G **[**GC**]** GC^{237}-GAC
**AD17** *proP* [281] ins (T)	ACC-TA **[**T**]** T^{281}-ATG
**AD18** *proP* [100] Δ(G)	CTC-GCT^{100} → CTC-Δ(G)CT^{100}
**AD19** *proP*::IS*1* [298]	GAA-GAC-CAC-GGG^{298} **[**GTA..**IS*1***..GAA**]** GAC-CAC-GGG-GTG
**AD20** *proP* [F380/S]	TTC^{380}/ TCC
**AD22** *proP* [299] Δ (G)	GTG-CTG^{299} → GTG-CTΔ(G)^{299}

[a]The identified mutations and the insertion site of an IS*1* element are located within the *proP* gene [11]. Each of these strains is derived from mutants carrying changes in the *proU* operon (*proV–proW–proX*). Otherwise, the genetic background of these strains corresponds to that of the the ProP^{+} ProU^{+} parent strain MC4100. The affected codons in the *proP* gene, either carrying mutations or hosting an IS*1*- insertion elements, are indicated. Duplicated sequences flanking the IS*1* insertion site are underlined. Ins: insertion; Δ: deletion.

Table S3. DNA-primers used for the amplification of the *proP* gene and of the *proU* operon and for the DNA-sequence analysis of these genes

Name	Sequence 5'- 3'	Purpose
ProP-for	CACTGGTAGGGTAAAAAGGTC	Amplification of *proP*
ProP-rev	GTATGCGCGTCAGAGATTAAG	Amplification of *proP*
ProP-Seq	AAGCGAACTTCCTCGATTGG	Sequencing of *proP*
ProU-for	AGGGGTTGCCTCAGATTCTCAG	Amplification of *proU* operon
ProU-rev	TTGTAGGCATGATAAGACGCGCC	Amplification of *proU* operon
ProU-Seq1	GCGACCGAATTGCCATTATGCA	Sequencing of *proU* operon
ProU-Seq2	GGTGGCGATTATCGTTTTCGCT	Sequencing of *proU* operon
ProU-Seq3	ACAGCGTTTGCCACGCTTAT	Sequencing of *proU* operon

REFERENCES

1. Haardt, M., et al., *The osmoprotectant proline betaine is a major substrate for the binding-protein-dependent transport system ProU of Escherichia coli K-12.* Mol Gen Genet, 1995. **246**(6): p. 783-786.
2. Ferenci, T., et al., *Genomic sequencing reveals regulatory mutations and recombinational events in the widely used MC4100 lineage of Escherichia coli K-12.* J Bacteriol, 2009. **191**(12): p. 4025-4029.
3. Cosquer, A., et al., *Antibacterial activity of glycine betaine analogues: involvement of osmoporters.* Bioorg Med Chem Lett, 2004. **14**(9): p. 2061-2065.
4. Bashir, A., et al., *Dimethlyglycine provides salt and temperature stress protection to Bacillus subtilis.* Appl Environ Microbiol, 2014. **80**(9): p. 2773-2785.
5. Lucht, J.M. and E. Bremer, *Adaptation of Escherichia coli to high osmolarity environments: osmoregulation of the high-affinity glycine betaine transport system ProU.* FEMS microbiology reviews, 1994. **14**(1): p. 3-20.
6. Wood, J.M., et al., *Osmosensing and osmoregulatory compatible solute accumulation by bacteria.* Comparative biochemistry and physiology. Part A, Molecular & integrative physiology, 2001. **130**(3): p. 437-460.
7. Bay, D.C. and R.J. Turner, *Small multidrug resistance protein EmrE reduces host pH and osmotic tolerance to metabolic quaternary cation osmoprotectants.* J Bacteriol, 2012. **194**(21): p. 5941-5948.
8. Moore, J.L., Taylor, S.M., Soloshonok, V.A., *An efficient and operationally convenient general synthesis of tertiary amines by direct alkylation of secondary amines with alkyl halides in the presence of Huenig's base.* ARKIVOC, 2005. **vi**: p. 287-292.
9. Gowrishankar, J., *Nucleotide-sequence of the osmoregulatory proU operon of Escherichia coli.* Journal of Bacteriology, 1989. **171**(4): p. 1923-1931.
10. Faatz, E., A. Middendorf, and E. Bremer, *Cloned structural genes for the osmotically regulated binding-protein-dependent glycine betaine transport-system (ProU) of Escherichia coli K-12.* Molecular Microbiology, 1988. **2**(2): p. 265-279.
11. Culham, D.E., et al., *Isolation and sequencing of Escherichia coli gene proP reveals unusual structural features of the osmoregulatory proline/betaine transporter, ProP.* J Mol Biol, 1993. **229**(1): p. 268-76.
